# Novel Viral DNA Polymerases From Metagenomes Suggest Genomic Sources of Strand-Displacing Biochemical Phenotypes

**DOI:** 10.3389/fmicb.2022.858366

**Published:** 2022-04-21

**Authors:** Rachel A. Keown, Jacob T. Dums, Phillip J. Brumm, Joyanne MacDonald, David A. Mead, Barbra D. Ferrell, Ryan M. Moore, Amelia O. Harrison, Shawn W. Polson, K. Eric Wommack

**Affiliations:** ^1^Department of Biological Sciences, College of Arts and Sciences, University of Delaware, Newark, DE, United States; ^2^Biotechnology Program, North Carolina State University, Raleigh, NC, United States; ^3^Varigen Biosciences Corporation, Middleton, WI, United States; ^4^Department of Plant and Soil Sciences, College of Agriculture and Natural Resources, University of Delaware, Newark, DE, United States; ^5^Center for Bioinformatics and Computational Biology, University of Delaware, Newark, DE, United States; ^6^Department of Computer and Information Sciences, College of Arts and Sciences, University of Delaware, Newark, DE, United States

**Keywords:** strand displacement, functional metagenomics, genome replication, virus, bacteriophage, enzymology

## Abstract

Viruses are the most abundant and diverse biological entities on the planet and constitute a significant proportion of Earth’s genetic diversity. Most of this diversity is not represented by isolated viral-host systems and has only been observed through sequencing of viral metagenomes (viromes) from environmental samples. Viromes provide snapshots of viral genetic potential, and a wealth of information on viral community ecology. These data also provide opportunities for exploring the biochemistry of novel viral enzymes. The *in vitro* biochemical characteristics of novel viral DNA polymerases were explored, testing hypothesized differences in polymerase biochemistry according to protein sequence phylogeny. Forty-eight viral DNA Polymerase I (PolA) proteins from estuarine viromes, hot spring metagenomes, and reference viruses, encompassing a broad representation of currently known diversity, were synthesized, expressed, and purified. Novel functionality was shown in multiple PolAs. Intriguingly, some of the estuarine viral polymerases demonstrated moderate to strong innate DNA strand displacement activity at high enzyme concentration. Strand-displacing polymerases have important technological applications where isothermal reactions are desirable. Bioinformatic investigation of genes neighboring these strand displacing polymerases found associations with SNF2 helicase-associated proteins. The specific function of SNF2 family enzymes is unknown for prokaryotes and viruses. In eukaryotes, SNF2 enzymes have chromatin remodeling functions but do not separate nucleic acid strands. This suggests the strand separation function may be fulfilled by the DNA polymerase for viruses carrying SNF2 helicase-associated proteins. Biochemical data elucidated from this study expands understanding of the biology and ecological behavior of unknown viruses. Moreover, given the numerous biotechnological applications of viral DNA polymerases, novel viral polymerases discovered within viromes may be a rich source of biological material for further *in vitro* DNA amplification advancements.

## Introduction

The foundations of today’s $105 billion United States biotechnology industry (Industry Market Research Reports and Statistics, 2021) are built upon fundamental discoveries in microbial and bacteriophage biology and nucleic acid biochemistry (Salmond and Fineran, 2015). While the *Thermus aquaticus* (Brock and Freeze, 1969) bacterial Family A DNA polymerase revolutionized molecular biology by enabling exponential *in vitro* amplification of a DNA template (Mullis et al., 1986), it has been bacteriophage DNA polymerases that have played a particularly outsized role in the development and advancement of DNA sequencing. The DNA polymerase of coliphage T7 overtook the Klenow fragment of *Escherichia coli* as a sequencing enzyme because of its unusual ability to incorporate strand-terminating dideoxynucleotides into the growing DNA strand (Tabor and Richardson, 1987, 1995). The DNA polymerase from Bacillus phage phi29 has the unusual property of strand displacement (Blanco and Salas, 1984), and has been critical in forensics and environmental science as this enzyme produces large quantities of DNA from minute amounts of starting template DNA. A thermostable DNA polymerase discovered from a viral metagenome (virome) enables single enzyme RT-PCR assays (PyroPhage, Lucigen Corporation, Moser et al., 2012), where previously this reaction was performed in two enzymatic steps requiring a reverse transcriptase followed by a thermostable DNA polymerase. Recent functional metagenomic approaches from hot spring environments have discovered high-fidelity phage DNA polymerases for PCR (Palmer et al., 2020). Our understanding of phage polymerase biochemistry is limited to these few examples, meanwhile there exists a significant amount of untapped potential in the virosphere (Schoenfeld et al., 2010).

Family A DNA polymerase (*polA*) is found in ca. 25% of double-stranded DNA (dsDNA) bacterial viruses (phages) (Wommack et al., 2015). While also found in bacteria, where it often plays an accessory role (e.g., *E. coli*), PolA is the primary enzyme used in phage genome replication. Phage *polA* genes have been identified in diverse environments including freshwater, marine, hot springs, and soils (Nasko et al., 2018; Palmer et al., 2020) and across a broad range of viral taxa (Weigel and Seitz, 2006). Previous *in vitro* mutagenesis studies of *polA* in *E. coli* phage T7 have shown that amino acid substitutions at residue 762 (*E. coli* numbering) change the *in vitro* biochemistry of the enzyme (Tabor and Richardson, 1987, 1995; Suzuki et al., 2000). A tyrosine substitution (Tyr762) of the wild type phenylalanine resulted in a highly processive *E. coli* polymerase with faster incorporation of deoxynucleotides (dNTPs, Astatke et al., 1998), while a leucine substitution (Leu762) produced a slower but more accurate Taq polymerase (Suzuki et al., 2000). While these mutagenesis studies are intriguing, they may not represent the true *in vivo* biochemistry of PolA proteins that natively carry the Phe762, Tyr762, and Leu762 variants, all of which are highly abundant in viromes. In fact, phylogenetic analysis indicates that viral PolAs diverge substantially from cellular PolAs which almost universally carry Phe762. Intriguingly, these examinations of PolA 762 variants indicated a possible connection between this variation and either a lytic (Phe and Tyr) or lysogenic (Leu) phage life cycle (Schmidt et al., 2014; Nasko et al., 2018). Further studies of this PolA to phage life cycle connection have been limited by lack of tractable host-virus systems. For example, no lytic viruses carrying a Tyr762 polymerase have been mutated to Leu762 to assess the impact of 762 variation on either polymerase biochemistry or the phage’s life cycle characteristics. It is unclear whether this single mutation will influence the infection dynamics of the phage, or if it could impact the co-functioning of other proteins involved in phage genome replication.

Most known viral diversity on Earth has been discovered from metagenomic studies (Nooij et al., 2018), as most viruses cannot be studied in laboratory isolation due to the lack of a cultivable host (Staley and Konopka, 1985; Sanjuán et al., 2021). Using shotgun viral metagenomics, researchers can capture a relatively unbiased snapshot of viral populations within environmental samples. While metagenomics has revealed much about unknown viral genetic diversity, understanding of the phenotypic characteristics existing within the abundance of newly discovered viruses has been limited, especially since obtaining the phenotypic characteristics of viruses is notoriously difficult (DeLong et al., 2021). This functional metagenomics study addressed this knowledge gap for unknown viruses by examining the *in vitro* biochemical characteristics of a diverse range of viral PolA enzymes from unknown environmental viruses.

## Materials and Methods

### DNA Polymerase I Diversity

The following databases were queried for putative DNA polymerase I peptide sequences: Viral RefSeq (Release 209, O’Leary et al., 2015), Joint Genome Institute’s IMG/VR (version 3, Roux et al., 2020), Global Ocean Viromes (GOV, Roux et al., 2016), and the Smithsonian Environmental Research Center (SERC) virome (Nasko et al., 2018) using target sequences of proteins belonging to the UniProt DNA polymerase type-A family (Version 2021_04; 53,102 sequences, UniProt Consortium, 2021). For all homology searches, MMseqs2 easy-search (version 13-45111, Steinegger and Söding, 2017) was used. Settings for homology searches were chosen depending on query data set size. For protein sequences from IMG/VR the following settings were used: –num-iterations 1, –start-sens 1, –sens-steps 3, -s 6 (sensitivity), –max-accept 1; while all other query sets used: –num-iterations 2, –start-sens 1, –sens-steps 3, -s 7 (sensitivity), –max-accept 1. The max-accept 1 option was used to recover any query protein sequence with a significant hit to the target database. All other settings were the default.

To filter sequence hits, any query sequence hitting a UniProt DNA polymerase type-A family in the homology search was checked for conserved residues with Protein Active Site Validation (PASV, version 2.0.1, Moore et al., 2021), in multiple sequence alignment (MSA) mode using Clustal Omega version 1.2.4 (default settings), and *E. coli* DNA polymerase I, strain K12 (UniProt acc. P00582) as the reference sequence. Any query sequence containing R688, D705, K758, and F, L, or Y at 762 (*E. coli* numbering) were retained for clustering.

The protein sequences that passed the PASV screen were then clustered. To avoid combining sequences from different source databases or different 762 position residues, clustering was performed within each database and within each 762 group (F,L,Y) at 75% identity over 80% of the sequence length using the easy-cluster function of MMseqs2 (version 13-45111) with the following settings: –cluster-reassign, –min-seq-id 0.75, -c 0.8, –cov-mode 0 (coverage of query and target). Cluster representatives were annotated with Reverse Position Specific BLAST (RPS-BLAST, version 2.11.0+). All domain models included in the NCBI CD-Search tool’s default “cdd” database (Lu et al., 2020)^1^ were used as the target database for the search. Sequences that were at least 400 AAs long and with at least one significant hit (*e*-value 1e-10) greater than or equal to 325 AAs to a conserved domain model that is a member of the DNA_pol_A Superfamily (acc. cl02626) were retained. To reduce the total number of sequences to a more manageable number for alignment and tree construction, cluster representatives from GOV and IMG/VR were subsampled to 225 sequences within each database-762 position pair (ex. 225 from GOV-F762, 225 from GOV-L762, etc.).

### Sequence Selection for DNA Polymerase Synthesis

**FIGURE 1:**
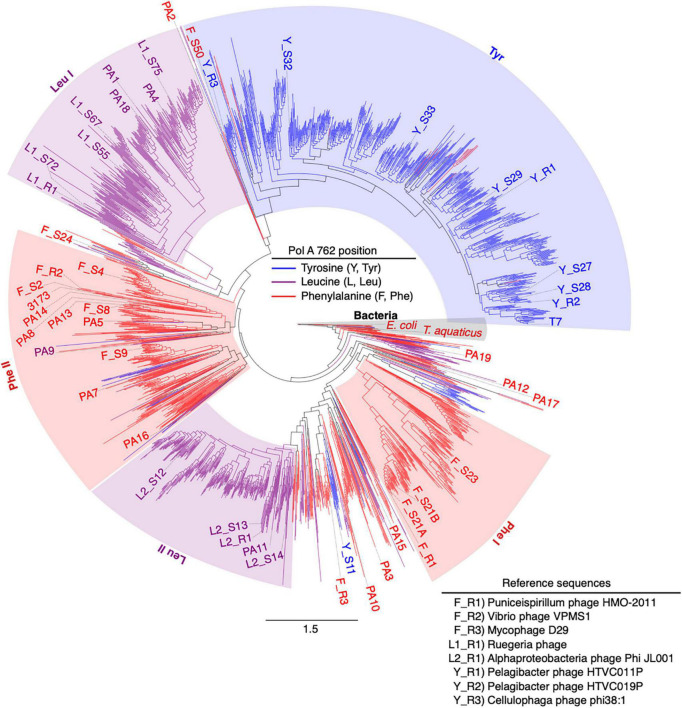
Synthesized family A DNA polymerase sequences span phylogenetic diversity. Approximate maximum likelihood tree of 2,130 PolA amino acid sequences trimmed to the region of interest (DNA_pol_A Superfamily, acc. cl02626), the polymerase domain. Branch coloring indicates residue identity at the 762 position within the protein sequence. Synthesized reference and virome sequences are labeled by 762 residue monophyletic clades [leucine, Leu I or Leu II; tyrosine, Tyr; (Nasko et al., 2018)] with two additional paraphyletic groups identified in this analysis (phenylalanine, Phe I or Phe II). Phe I and Phe II. Bacterial reference sequences clustered toward the root of the tree are shaded in gray. All synthesized sequences are labeled by source (reference, R; virome, S; or IMG/M database, PA). PolA with previously published biochemical characterization are labeled separately (T7, *E. coli*, 3173, and *T. aquaticus*). Scale bar represents the number of amino acid substitutions per site.

In total, 48 PolA sequences were selected for synthesis. Eighteen phage-like PolA sequences from microbial metagenomes of hot spring environments were selected from the Joint Genome Institute’s IMG/M database (Chen et al., 2021; Mukherjee et al., 2021) for potential biotechnology interests. Thirty additional PolA sequences were chosen with a goal of representing a broad range of PolA viral phylogenetic diversity (Figure 1). Of these thirty, seven PolA sequences distributed across five PolA clades and groups were selected from Viral RefSeq genome-sequenced, cultivated reference phages. One PolA sequence from an unpublished phage genome was selected (L1_R1 PolA of Rugeria phage 67) providing diversity coverage that was otherwise missing. The remaining 22 PolA sequences were selected from the SERC virome library contigs (NCBI GCA_002237165.1, Marine et al., 2017; Nasko et al., 2018). With one exception (F_S2), all SERC PolA sequences occurred on virome contigs of >10 kb in length.

### Open Reading Frame Annotation

Contigs containing selected PolAs were examined for adjacent gene sequences with special attention to helicase sequences (Supplementary Table S1). Virome-sourced contigs and the unpublished phage genome (Rugeria phage 67) were examined using MetaGeneAnnotator (Noguchi et al., 2008). Resulting open reading frames (ORFs) were subject to a protein blast (blastp, Geneious v10.2.6^2^) against the UniProt database (Version 2021_04; UniProt Consortium, 2021) and the top result was noted. All other genome-sequenced (reference) virus gene annotations were obtained from NCBI. Microbial metagenome annotations were obtained from the IMG/M database.

### Alignments and Phylogenetic Trees

For visualization of PolA phylogenetic diversity, biochemically characterized PolA amino acid sequences were manually retrieved from UniProt and NCBI and include those from *Escherichia coli* strain K12 (acc. P00582), *Thermus aquaticus* (Taq, acc. P19821), Bacteriophage T7 (acc. P00581), and PyroPhage 3173 (3173, acc. AADL99605.1). In addition, 33 bacterial and nine viral amino acid reference sequences were retained from the original target sequences collected from UniProt (belonging to the DNA polymerase type-A family, reviewed non-eukaryotic members only) for tree building. Accession numbers for these sequences are listed in Supplementary Table S2 (46 amino acid sequences total). Final sequence numbers used in tree building are as follows: 36 bacterial SwissProt, 9 viral SwissProt, 1 NCBI (3173), 18 IMG/M (synthesized), 263 Viral RefSeq, 675 IMG/VR, 675 GOV, and 453 SERC (including 22 synthesized sequences). DNA_pol_A Superfamily (acc. cl02626) annotations were extracted using Geneious v10.2.6 from all full-length sequences. When multiple significant hits to this accession were present, the longest hit was selected for downstream alignment and tree building.

All 2,130 PolA sequences identified from the virome and curated viral databases were aligned in Geneious v10.2.6 with MAFFT v7.450 plugin using the FFT-NS-i x 1000 algorithm and BLOSUM62 scoring matrix (Katoh and Standley, 2013). An approximate maximum likelihood tree of trimmed PolA sequences was constructed in Geneious using FastTree version 2.1.11 (Price et al., 2010) with default settings, and rooted on the *E. coli* sequence using the root function. Tree branches, leaf nodes, and reference sequence labels were annotated by 762-type using Iroki (Moore et al., 2020). The 762 position identity was manually confirmed by alignment to the *E. coli* reference sequence.

The 48 sequences selected for synthesis and four biochemically characterized references (*E. coli*, T7, Taq, and 3173) were aligned using Geneious v10.2.6 MAFFT v7.450 plugin using the G-INS-i mode with default options (scoring matrix: BLOSUM62, gap open 1.53, offset value 0.123). A maximum likelihood tree was inferred from this alignment using IQ-Tree version 1.6.12 (Nguyen et al., 2014) using ultrafast bootstrap approximation with 1,000 replicates (UFBoot, Hoang et al., 2017), Shimodaira-Hasegawa approximate likelihood ratio test with 1000 bootstrap replicates (SH-aLRT, Guindon et al., 2010), and hill-climbing nearest neighbor interchange optimization of UFBoot trees (flag -bnni). A best-fit model was automatically selected using IQ-Tree’s ModelFinder algorithm (Kalyaanamoorthy et al., 2017). Tree branches, leaf nodes, sequence labels, and bars denoting biochemical data, environment of origin, data source, and helicase neighbor were annotated using Iroki (Moore et al., 2020).

### Protein Production

#### Maltose-Binding Protein Fusion Products

Protein sequences were reverse translated and codon-optimized for *E. coli* expression with a TEV cleavage sequence fused to the N-terminus and flanked by a *Bam*HI site at the 5′ end and a *Not*I site at the 3′ end (Supplementary Table S3). Synthesized sequences were cloned in between the *Bam*HI and *Not*I sites of an arabinose inducible pD871-based vector containing an N-terminal 6xHis tag and maltose binding protein. DNA synthesis and cloning were performed under a Department of Energy Joint Genome Institute Synthesis project (FY17). Clones were grown and cells were harvested and lysed as described below. Subsequently, His-tagged proteins with an MBP fusion were purified using a Ni column and dialysis as described below. Regrettably, purified MBP fusion proteins were inactive when assayed for primer extension, therefore this necessitated MBP removal from the original synthesized sequences (details below).

#### Truncated 6xHis-Tagged Protein Products

The MBP sequence was removed from vectors by amplifying the vector without the MBP and TEV cleavage site using primers with overlaps to join the 6xHis tag directly to the polymerase sequence. Amplified vectors were circularized by DNA ligase and constructs were confirmed by sequencing and transformed into E. cloni^®^ 10G chemically competent cells (Lucigen Corporation, Middleton, WI, United States). Cultures were grown overnight on LB plates with 30 μg/mL kanamycin. Four colonies were chosen per clone and replicate cultures grown overnight in LB media. Minipreps were performed using the ZymoPURE Plasmid Miniprep Kit (Zymo Research, Irvine, CA, United States). Plasmid sizes were confirmed by *Nco*I-HF Restriction Endonuclease digests (New England Biolabs, Ipswich, MA, United States).

Cultures were inoculated from glycerol stocks in 100 mL of LB media with 0.4% dextrose (w/v) and 30 μg/mL kanamycin and grown overnight at 37°C and 200 rpm. The overnight culture was divided into two 1 L volumes of LB with 0.4% (w/v) rhamnose and 30 μg/mL kanamycin and grown overnight at 24°C and 200 rpm. Cells were pelleted by centrifugation for 30 min at 3,220 × *g* and pellets were resuspended in 10 volumes of lytic wash buffer (100 mM Tris–HCl pH 8, 250 mM NaCl, 30 mM imidazole). The suspension was sonicated on ice, 15 s on, 15 s off, for 15 min at amplitude 50, and clarified by centrifugation for 30 min at 11,952 × *g* and 4°C. The clarified lysate was loaded onto a 20 mL HisPur™ Ni-NTA Resin column pre-equilibrated with extraction buffer (100 mM Tris–HCl pH 8, 250 mM NaCl, 30 mM imidazole). The column was washed with ten column volumes of extraction buffer, and the polymerase eluted with ten column volumes of elution buffer (100 mM Tris–HCl pH 8, 250 mM NaCl, 300 mM imidazole) and collected in 6 mL fractions. Protein products within collected fractions were confirmed via SDS-PAGE using 4–20% Mini-PROTEAN TGX precast protein gels (Bio-Rad Laboratories, Hercules, CA, United States). Samples were mixed with 4X Laemmli protein sample buffer (Bio-Rad Laboratories) and incubated at 95°C for 5 min prior to gel loading. Electrophoresis was performed at 300 V for 15 min in Tris-base Glycine SDS running buffer. Gels were stained in 0.1% Coomassie R-250 (Research Products International, Mount Prospect, IL, United States), 40% ethanol, 10% acetic acid for 10 to 15 min, de-stained overnight in 10% ethanol, 7.5% acetic acid with subsequent washes in 7.5% acetic acid, and imaged.

The eluent was concentrated by dialysis with Cas9 storage buffer (50 mM Tris–HCl pH 7.5, 50 mM KCl, 1 mM DTT, 1 mM EDTA, 50% glycerol). The dialyzed protein product was confirmed by SDS-PAGE as previously described. Cas9 storage buffer protein stocks were used in all downstream assays.

### Primer Extension and Strand Displacement Assay

Primer extension assays were performed in 1X phi29 DNA Polymerase reaction buffer [50 mM Tris–HCl pH 7.5, 10 mM MgCl_2_, 10 mM (NH_4_)_2_SO_4_, 1 mM DTT] or 1X Bam35 reaction buffer (40 mM Tris–HCl pH 7.5, 1 mM MgCl_2_, 50 mM KCl, 0.50% Tween 20). Thirty microliter reactions included 400 ng M13mp18 single stranded phage DNA (Bayou Biolabs, Metairie, LA, United States), 3.3 μM ssM13 FT primer with three phosphonothioate bonds introduced at the 3′ end (5′- CGCCAGGGTTTTCCCAGTCAC*G*A*C -3′, Integrated DNA Technologies, Coralville, IA, United States), dNTPs at high (1 mM) or low (200 μM) concentrations, and seven 1:1 serial dilutions of an initial 5 μg protein/reaction concentration in 1X sample buffer. Reactions were incubated at 30°C with the thermocycler lid set to 55°C for 2 h, and then mixed with 6X green loading buffer (36% glycerol, 0.144% orange G, 0.012% xylene cyanol ff, 6X TAE, 0.48% SDS) and heat-killed for 2 min at 70°C. Products were visualized on a 0.7% agarose gel in 1X TAE buffer with 0.01% ethidium bromide run at 80 V for 1 h. A 15 μL reaction with phi29 DNA polymerase (Thermo Fisher Scientific) served as a positive control and was incubated at 37°C for 10 min and heat killed as described above. Assays with hot spring polymerases were performed as described above with a temperature modification of 65°C with the thermocycler lid set to 55°C.

The two strand displacing estuarine polymerases, F_S21A and F_S24, were subsequently used for comparing primer extension activity from M13mp18 single stranded phage DNA or double stranded M13mp18 plasmid (Bayou Biolabs) templates. Reactions were incubated for 18.5 h at 30°C with the lid set to 55°C, heat-killed for 10 min at 70°C with 6X green loading buffer and visualized on an agarose gel as described above.

### Exonuclease Activity Assay

All hot spring polymerases and the F_S21A and F_S24 estuarine polymerases were tested for exonuclease activity in 25 μL reactions with 50 ng phage λ DNA *Hin*dIII Digest (New England Biolabs), 2 mM dNTPs, and 5 μg protein/reaction in 1X phi29 reaction buffer. Reactions were incubated at 65°C (30°C for F_S21A and F_S24) for 2 h, heat-killed for 10 min at 70°C with 6X green loading buffer and visualized on an agarose gel as described above.

## Results

### DNA Polymerases Form Three Diverse Phylogenetic Clades and Two Polyphyletic Groups Largely Consistent With 762-Position Amino Acid Residue

**FIGURE 2:**
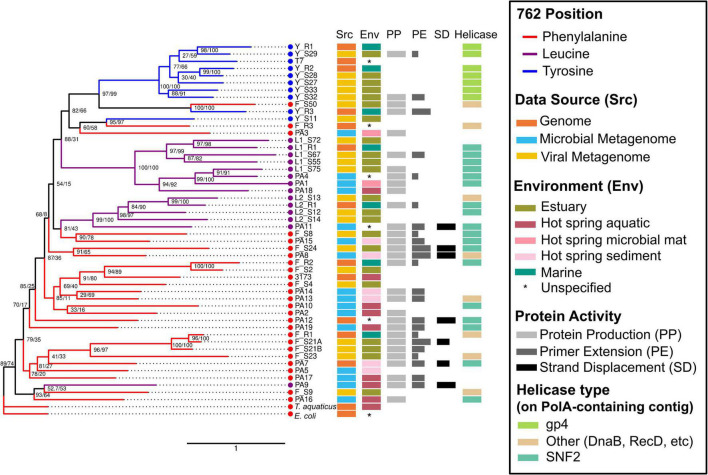
Synthesized family A DNA polymerase sequences produce proteins with diverse biochemistry. Maximum likelihood tree of four biochemically characterized references (*E. coli*, *T. aquaticus*, T7, and 3173) and 48 synthesized family A DNA polymerase amino acid sequences trimmed to the region of interest (DNA_pol_A Superfamily, acc. cl02626). Synthesized reference, viral metagenome (virome), and microbial metagenome sequences are labeled by 762 residue group (phenylalanine F) or clade (leucine, L1 or L2, or tyrosine, Y). The strength of primer extension (PE) and strand displacement (SD) activities are reflected in the bar lengths. Node values represent ultrafast bootstrap support (UFBoot) and Shimodaira-Hasegawa approximate likelihood ratio test (SH-aLRT), respectively. Scale bar represents the number of amino acid substitutions per site.

Phylogenetic analysis of Family A DNA polymerase sequences from viromes and sequenced reference virus genomes indicated that the amino acid identity of the 762 position (*E. coli* numbering) correlated with PolA sequence phylogeny (Figure 1). In prior work, five major clades of viral PolA sequences were identified according to the 762 position (Nasko et al., 2018). With the addition of more than 1,500 viral PolA sequences, this analysis largely recapitulated previously identified clades, and expanded diversity in multiple groupings. The addition of 36 bacterial reference sequences, in addition to rooting the phylogeny on the *E. coli* sequence, reorganized the phenylalanine groupings from a previous report (Nasko et al., 2018). All bacterial sequences clustered together near the root. Previous viral PolA clade designations F1 and F2, while largely intact, are not supported as monophyletic clades and as such are organized here as paraphyletic groups Phe I and Phe II, respectively. These groups remain largely homogeneous in their 762 identity aside from a few deep-branching sequences containing Leu762 and Tyr762 residues. In the large tyrosine clade (Tyr) a few shallow-branching Phe sequences were detected. The root of the Tyr clade contained a mix of deep-branching Phe sequences with more shallow-branching Tyr sequences, containing both characterized sequences of Y_R3 and F_S50. Adjacent to the Tyr clade were a small number of deep-branching Phe sequences that contained one characterized representative (PA2). The Leu I, Leu II, and Tyr clades remained stable with high levels of bootstrap support (Figure 2).

The Phe II group contained the most sequences (12) with either attempted or successful *in vitro* characterization of polymerase biochemistry. Of the remaining synthesized sequences, eight belonged to the Leu I clade, nine to Tyr, five to Leu II, and four to the Phe I group. Ten characterized sequences did not fall into any of these groupings and were labeled outside of the color shading (Figure 1).

### Metagenome and Virome-Derived Enzymes Exhibit Variable Levels of Protein Expression and Purification

Proteins predicted from 48 synthesized gene sequences included 26 Phe762, 14 Leu762, and nine Tyr762; were 564 to 760 amino acids long; and ranged in predicted isoelectric point from 5.1–9.2 (Supplementary Table S3).

Fusion proteins with MBP-TEV tags were successfully produced and purified for all selected clones (Supplementary Figure S1). TEV protease cleavage of the maltose-binding protein fusion was unsuccessful for all polymerases. Despite MBP’s broad success in producing and purifying soluble proteins, none of these proteins demonstrated *in vitro* primer extension. This necessitated a change in approach and removal of the MBP fusion protein and TEV cleavage site from the synthesized PolA gene sequences to include only the 6xHis tag for purification.

**FIGURE 3:**
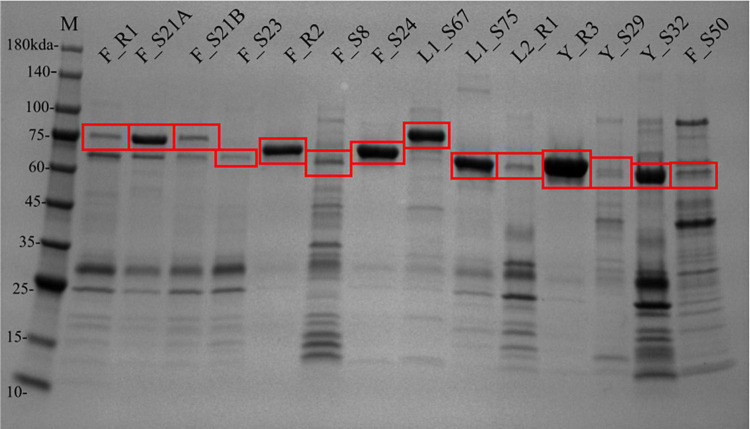
Protein production from synthesized reference and virome sequences. SDS-PAGE gel of 6xHis-tagged proteins from reference and virome clones. Red boxes indicate protein bands of interest (expected PolA size ∼64–75 kDa). Prefix of clone names indicate the PolA 762 residue and clade (Figure 1): phenylalanine group (F), leucine clades (L1 or L2), or tyrosine clade (Y); and the character following the underscore indicates the source: reference sequence (R) or virome sequence (S).

After the removal of MBP-TEV tags, 48 sequences were successfully cloned. Sequence Y_S28 was not successfully cloned with the truncated 6xHis tag. Thirty-one of 47 synthesized and cloned gene sequences (65%) produced soluble His-tagged proteins of the approximate expected size (Figures 2, 3 and Supplementar Table S4). Protein production was distributed across all PolA phylogenetic clades. PolAs having a Phe762 had the greatest success rate, 77% (20 of 26), followed by Leu762, 57% (8 of 14), and lastly Tyr762, 33% (3 of 9). Active soluble proteins were successfully produced from sequences derived from a variety of sources and environments. The greatest success rate according to data source was microbial metagenomes where 88% (15 of 17) of cloned PolAs produced protein, followed by genome-sequenced phages at 55% (5 of 9). PolAs from viromes had more modest success rates of 45% (10 of 22). There was a stark difference in protein production success according to environment. Cloned PolAs from hot spring environments had an 81% (13 of 16) production success rate. The success rate for estuarine virome PolAs was half that of hot springs, 43% (9 of 21). While some protein fractions, such as those from clones Y_R3, F_R2, and F_S24, provided nearly pure preparations of PolA, others such as F_S8 and F_S50 contained dozens of additional contaminating proteins (Figure 3). Regardless of the level of purity, these fractions were used in subsequent biochemical characterization assays.

### Primer Extension and Strand Displacement Activity Observed in Viral Polymerase I Clones

Of the 30 clones producing proteins, 23 provided some level of *in vitro* primer extension of M13mp18 single stranded phage DNA template. While expression and purification success of Tyr762 PolAs was low, all of these polymerases demonstrated *in vitro* primer extension with no capability of strand displacement (Figure 2). The success of Phe762 PolAs in expression and purification was matched by success in primer extension assays (78%, 15 of 19). Surprisingly, 33% (5 of 15) of these primer-extending Phe762 PolAs also demonstrated strand displacement activity. While Leu762 PolAs showed slightly less primer extension success (63%, 5 of 8), like Phe762, 40% (2 of 5) of these primer-extending Leu762 PolAs showed strand displacement activity (Figure 2).

**FIGURE 4:**
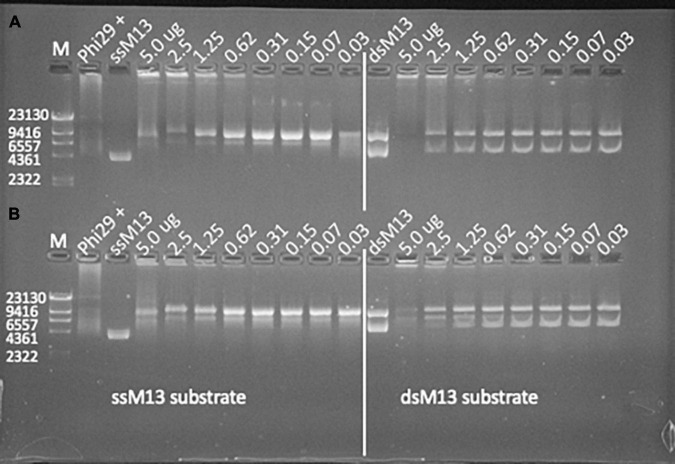
*In vitro* primer extension and strand displacement in two synthesized virome polymerases. **(A)** F_S24 and **(B)** F_S21A at decreasing (left to right) concentrations (μg/reaction) in 1X phi29 buffer after 18.5 h incubation at 30°C. Both polymerases demonstrated primer extension and strand displacement from both single-stranded M13 (left) and double-stranded M13 (right) DNA templates. Strand displacement is evident in the phi29 DNA polymerase positive control after 10 min incubation at 37°C.

Polymerase I proteins synthesized and successfully produced from genome sequenced viruses demonstrated the greatest amount of *in vitro* biochemical activity as all five of the produced proteins demonstrated some level of primer extension activity and one demonstrated strand displacement activity. Of the virome data sources, 80% (8 of 10) of the produced PolA proteins demonstrated *in vitro* primer extension activity. Two Phe762 virome proteins, F_S21A and F_S24, also demonstrated moderate to strong innate strand displacement activity with both ssM13 and dsM13 DNA templates in 1X phi29 buffer after an 18.5 h incubation (Figure 4). Of the metagenome derived proteins, F_S21A and F_S24 preparations were relatively pure, although several smaller proteins cooccurred with the PolA target protein in the isolated fraction (Figure 3). Of these two proteins, F_S24 had higher purity and demonstrated stronger activity with strand displacement evident down to 0.3 μg of protein with either template. Produced PolAs from microbial metagenome sources were the least successful in primer extension with 67% (10 of 15), and three showing strand displacement capability. None of the produced and tested PolAs demonstrated detectable levels of *in vitro* exonuclease activity (data not shown).

Co-purified proteins were present at moderate to high levels in several virome protein stocks (Figure 3). It is possible that co-purified proteins could have contributed to the observed primer extension activity. However, it is equally likely that the co-purified proteins were truncated forms of the target polymerase sequence given their affinity for the nickel chromatography column. The co-purified proteins were not likely of *E. coli* origin due to the size variation and intensity differences across the protein purifications. The most active strand-displacing virome sequences were among the better purified stocks (F_S21A and F_S24). Moreover, the *E. coli* strain used for protein production does not carry genes for innate strand-displacing DNA polymerases, therefore we are confident that the observed strand displacement activity came from the cloned phage PolA sequence. Nonetheless, future quantitative assays of biochemical activity will require removal of co-purified proteins.

### Viral Polymerase I–Helicase Association Patterns by 762 Residue Identity

Adjacent gene sequence annotations were considered for all 48 synthesized PolA sequences (Supplementary Table S1), with special focus given to associated helicase sequences. Seven of nine (78%) Tyr762 PolAs synthesized in this study were proximal to a ring-shaped gp4 helicase on their source contig (Figure 2), while the remaining two had no recognizable helicase annotation. None of the Phe762 or Leu762 PolA-containing contigs carried a gp4 type helicase. Nine of fourteen (64%) Leu762 PolA-containing contigs carried an SNF2-like helicase, while one had a RecB/D type helicase. Four of fourteen (28.5%) Leu762 PolAs did not have an adjacent helicase annotation possibly due to shorter contig length. The remaining Phe762 PolAs were more variable in helicase association. Nine of twenty-five (36%) encoded an SNF2 annotation, seven (28%) had DnaB, and two (8%) encoded both SNF2 and DnaB. The remaining nine (36%) Phe762 PolAs had no helicase annotation present. Additional replication-related genes and structural genes are noted in Supplementary Table S1.

## Discussion

### Functional Metagenomics as a Tool for Genome to Phenome Investigations in Viruses of Microbes

A common rationale for functional metagenomics investigations has been the discovery of novel biochemical characteristics in microbial proteins (Ufarté et al., 2015). Addressing needs for improved cellulases in biofuel production (Banu et al., 2021), discovery of novel antimicrobials for therapeutic needs (Gillespie et al., 2002), improved food processing reactions in agriculture (Richardson et al., 2002), and novel pre- and probiotics for improved human gut microbiome health (Wang et al., 2012; Guazzaroni et al., 2013) have all driven studies utilizing the cloning of environmental DNA followed by *in vitro* expression and screening for a particular biochemical activity of interest.

This functional metagenomics study took a different approach, relying on specific hypotheses of structure-function relationships that shape enzyme kinetics (in this case DNA polymerase A) as an experimental framework. Bioinformatic identification and phylogenetic analyses of novel protein sequences (viral PolAs, Figure 1) was used for rational selection of a broad cross-section of genes testing hypothesized connections between PolA 762 identity and enzyme biochemistry. Selected synthesized gene sequences from both metagenomic and genomic source data were cloned, expressed, and tested for their biochemical phenotypes. Prior investigations of PolA proteins within viromes demonstrated that Tyr762 and Leu762 residues were as common as the cellular wild type Phe762 residue within the virioplankton (Nasko et al., 2018). In some environments, such as the Chesapeake Bay, virioplankton populations with Leu762 predominated over those with Tyr762 or Phe762 PolAs, possibly indicating an abundance of temperate bacteriophages within Chesapeake Bay virioplankton (Schmidt et al., 2014). Virome data have also shown that Phe762 or Tyr762 PolA frequently demonstrate genomic associations that would be favored by a virulent phage requiring rapid and unrestrained levels of DNA synthesis for viral replication (e.g., associations with ribonucleotide reductases (RNRs) and highly processive superfamily IV gp4 ringed helicases) (Dwivedi et al., 2013; Sakowski et al., 2014; Schmidt et al., 2014; Iranzo et al., 2016; Nasko et al., 2018). In contrast, Leu762 PolAs which demonstrate slower but higher fidelity *in vitro* DNA replication (Suzuki et al., 2000) (kinetics hypothesized to be favorable to lysogenic phages) rarely showed such genome associations (Nasko et al., 2018). Outside of mutagenized PolA studies in *E. coli* and coliphage T7 PolA (Tabor and Richardson, 1987, 1995; Suzuki et al., 2000), little is known of the biochemical diversity among PolA enzymes within viruses. This study expanded biochemical characterization of diverse viral PolA enzymes seeking data on how biochemical changes within viral enzymes might influence viral-host interactions in nature. Ideally, such connections should be studied using cultivated phages, however, as our phylogenetic analysis of viral and virome PolA proteins demonstrates (Figure 1) cultivated phages poorly represent the vast diversity of viruses seen within metagenomes (Breitbart et al., 2002; Mizuno et al., 2013). Understanding the limitations of studying PolA enzymes from only cultivated phages, we included *polA* genes observed within shotgun viral metagenomes for a directed functional metagenomics approach examining whether position 762 identity influenced PolA biochemical phenotypes in polymerases from unknown environmental viruses.

### Genome Associations Align With Polymerase I Biochemistry

Eighty percent of the expressed PolAs demonstrated some level of *in vitro* primer extension activity indicating that viral PolAs were robust candidates for functional metagenomic exploration. Primer extension failure was restricted to the Leu762 and Phe762 PolAs (Figure 2). It is an intriguing proposition that the hypothesized prevalence of Tyr762 PolA in virulent phage reflects the durability of these PolAs for *in vitro* primer extension (all three Tyr762 PolAs that expressed showed primer extension). The commercial success of the coliphage T7 PolA for *in vitro* DNA synthesis also supports this idea (Zhu, 2014). Conversely, only Phe762 and Leu762 PolA proteins showed *in vitro* strand displacement activity. In the light of our hypothesized genome to phenome connection between PolA position 762 identities and phage life cycle it makes sense that only Leu762 and Phe762 PolAs demonstrated strand displacement activity. Highly virulent phages require rapid DNA synthesis for replicating as many virus particles as possible in the shortest amount of time (Benkovic and Spiering, 2017). For example, many T7-like cyanophages (infecting cyanobacteria) that carry PolA will synthesize more DNA than is present in the bacterial host cell during the average infection cycle (Sullivan et al., 2005; Thompson et al., 2011, 2016). A strand displacing polymerase could restrict the speed and efficiency of DNA synthesis which could lengthen the infectious cycle, a possibly negative fitness consequence for a highly virulent phage.

Genes surrounding the viral DNA polymerase are commonly utilized in replication, as they are transcribed and translated in tandem during viral replication (Schmid et al., 2014). Consistent relationships between PolA and specific helicases have previously been observed within viromes (Nasko et al., 2018). Fast and processive ring shaped helicases such as DnaB and gp4 occurred predominantly with Phe762 and Tyr762 polymerases, respectively. In addition, these ringed helicases commonly accompanied an RNR gene on the same contig, suggesting a lytic life style. A gp4 helicase was encoded directly downstream of a Tyr762 PolA on 77% of selected virome contigs in this study (Figure 2), agreeing with prior observations (Nasko et al., 2018). The association frequency between gp4 helicase and Tyr762 PolA may have been even higher given the fragmentary nature of shotgun metagenome data. Nevertheless, the lack of strand displacement activity observed in the synthesized Tyr762 polymerases may reflect the fact that these polymerases typically associate with highly processive ringed helicases.

Genome associations between Leu762 and Phe762 polymerases and ring-shaped gp4-like helicases were not observed in the PA or reference sequences (Figures 1, 2) from this study nor in the other sequences also assessed in a prior virome study (Nasko et al., 2018). The lack of association with gp4 suggests that phages carrying Leu762 or Phe762 polymerases will replicate at a slower rate, limited by the rate at which their genomes are unwound. Leu762 polymerases occurred predominantly with non-ringed oligomeric helicases (the slower-moving counterpart to ring-shaped helicases) UvrD and RecB/D, and SNF2 helicase-associated proteins, and these contigs commonly lacked an RNR (Supplementary Table S1). This type of gene content is hypothesized for temperate or pseudo-temperate phages (Schmidt et al., 2014). The association of Phe762 polymerases and helicases was more varied than Tyr or Leu-type polymerases (Supplementary Table S1) possibly reflecting the unstable nature of the Phe762 phylogeny (Figures 1, 2). Another possible reason why a greater diversity of gene associations was observed among these polymerases is that Phe762 polymerases are known to occur in both lytic and temperate phages (Schmidt et al., 2014).

Helicases are classified by having three common abilities: nucleic acid binding by Walker A and Walker B domains (Walker et al., 1982), NTP binding, and NTP-driven unwinding of nucleic acids (Brosh and Matson, 2020). Enzymes that bind DNA but lack an ability to unwind or separate strands of DNA, are considered translocases (Enemark and Joshua-Tor, 2008), a protein class related to helicases. Helicase-like proteins named SNF2, a group within helicase Superfamily 2 (SF2), are defined by a tandem repeat of two RecA-like domains and seven additional helicase-related motifs (Eisen et al., 1995). Yet, SNF2 proteins are translocases, not helicases, as they cannot separate nucleic acid strands. SNF2 DNA translocases apply torsional strain to DNA, creating a force that remodels DNA-protein complexes such as histones in eukaryotes (Flaus et al., 2006). This mechanism is not fully understood in eukaryotes, and SNF2 annotations are commonly present in both prokaryotes and virus groups for which the function of this protein is still unknown (Ryan and Owen-Hughes, 2011). The observed predominance of associations between strand displacing polymerases and SNF2 translocases indicates that these polymerases may occur in viral genomes that do not contain other enzymes capable of separating DNA strands.

### Selection Pressure Fuels the Biochemical Diversity of Viral DNA Polymerases

Viral fitness directly depends on genome replication during infection. Thus, it logically follows that viral DNA replication systems are likely under intensive positive selection pressure for efficient replication. Some viruses carry many replication-related genes, while others rely to varying degrees on the host replication machinery. The genome size of dsDNA viruses correlates with the number of replication genes they carry (Kazlauskas et al., 2016). The contrasting evolutionary pressure on DNA replication systems between lytic and lysogenic dsDNA phages is apparent when comparing the DNA replication requirements of a lytic phage and a lysogenic phage and their *E. coli* host. Coliphage T7 with a genome of 39,937 bp, a burst size of ∼180 virions, and a latent period of 17 min replicates its dsDNA genome at ∼425 kb/min (Nguyen and Kang, 2014). Coliphage lambda (48,502 bp, ∼170 virions, and 51 min; Wu and Taylor, 1971; Wang, 2006) replicates its dsDNA genome at ∼161 kb/min, a rate 2.5X slower than T7. In contrast, the *E. coli* host for these two phages with a median genome size of 5,100 kb and a doubling time of 20 min replicates its genome at a slower rate of ∼255 kb/min. These estimated rates for phages T7 and lambda are conservative as phage dsDNA replication consumes only 80% of the latent period.

However, both T7 and lambda achieve these replication rates with fewer replisome proteins when compared to their host (Wu and Taylor, 1971; Dunn and Studier, 1983). In bacteria, DNA polymerase III (PolC), the primary holoenzyme for genome replication, consists of ten subunits and three functional molecules (McHenry, 1991). This large enzyme is highly processive, polymerizing up to 1,000 nucleotides/sec (Pomerantz and O’Donnell, 2007), yet its large size would be disadvantageous in a streamlined phage genome. In bacteria, DNA polymerase I (PolA) is responsible for DNA repair and removal of primers and polymerase II (PolB) provides proofreading (Cai et al., 1995; Blanco and Blanco, 2017). In contrast, among viruses DNA polymerases I and II are the most common and are the primary replicase enzymes for viral genome replication. Global computational analysis of replication genes in sequenced phages uncovered only eight occurrences of DNA polymerase III genes in 1,574 viruses (Kazlauskas et al., 2016). Thus, the phylogenetic diversity specifically observed for PolA within viromes (Figure 1) is not surprising given the prevalence and selective pressure on this DNA polymerase within viruses.

It is also clear that the sequence diversity in PolA is matched by functional changes to the enzyme in viruses, which may reflect an efficient use of genome space and expanded functionalities within single proteins. Within bacteria, PolA contains two exonuclease domains, one catalyzing excision in the 3′ to 5′ direction and the other in the 5′ to 3′ direction, the latter of which is responsible for primer removal of Okazaki fragments on the lagging strand (Makiela-Dzbenska et al., 2009). In viruses, the 5′ to 3′ exonuclease domain is commonly missing, leaving only the 3′ to 5′ domain and the polymerase domain, sometimes noted as the Klenow fragment (Kazlauskas et al., 2016). Therefore, different biochemical mechanisms not involving a 5′ to 3′ exonuclease domain on the DNA polymerase holoenzyme have evolved in viruses. In T7, primers are removed from Okazaki fragments by the nuclease action of protein gp6, encoded downstream of the polymerase (Hamdan and Richardson, 2009; Liu et al., 2017). While the *E. coli* 5′ to 3′ exonuclease is encoded in 323 amino acids (UniProt acc. P00582), T7’s comparably sized 300 amino acid gp6 protein (UniProt acc. P00638) performs additional functions including DNA packaging, host DNA degradation, and phage genetic recombination (Son and Serwer, 1992). In other cases, a function performed by accessory replication proteins, such as helicases (Frick and Lam, 2006), may be assumed by the polymerase. Our *in vitro* assays demonstrate that some viral PolAs, such as F_S21A and F_S24, may have evolved the capability of strand displacement possibly replacing helicase function in the replisome. It is interesting to note that the genome of *Cellulophaga* phage ϕ:38:1 (Holmfeldt et al., 2013), a genome-sequenced reference phage containing a Tyr762 PolA enzyme assessed in the study, does not contain an identifiable DNA helicase. While this PolA (Y_R3, Figure 1), like the other Tyr762 PolA proteins, did not exhibit strand displacement activity, it is possible that under different assay conditions strand displacement activity could be exhibited. Alternatively, it is possible that unwinding and strand displacement activities are performed by an unknown protein within the ϕ:38:1 genome or that this phage utilizes a helicase from its host for performing this essential function for DNA replication.

### Future Work

Using the design of experiments approach (DoE) should expedite future efforts toward testing assay optimization and ultimately quantitative measurements of PolA enzyme kinetics (Onyeogaziri and Papaneophytou, 2019). These additional efforts altering buffer composition, pH, temperature, and concentration of divalent cations may coax activity out of PolA proteins that failed primer extension in this study (Brooks et al., 2012). Higher purity preparations may improve activity for some clones, as co-purified proteins may have inhibited PolA activity. Sequential chromatography columns for optimized purifications can be used on a per-protein basis, considering the differences in size and predicted isoelectric point (pI) of proteins of interest in highly contaminated enzyme stocks (Kadonaga and Tjian, 1986). One alternative purification solution might be use of a cell-free transcription translation system (Khambhati et al., 2019). Recently, improved protocols for cell-free systems have been published addressing both production efficiency and cost of production (Wiegand et al., 2019). For example, cell-free systems produced more than 13,000 human proteins that would have otherwise been impossible to study *in vitro* (Goshima et al., 2008). Few functional metagenomics studies have used cell-free systems, but with the lowering price and increasing yield it may be a viable option for future studies.

## Data Availability Statement

The datasets presented in this study can be found online through Zenodo (https://doi.org/10.5281/zenodo.5826200) and UniProt (accessions listed in Supplementary Table S2).

## Author Contributions

KW, SP, DM, PB, and JD designed the research. PB, JM, RK, and BF purified and characterized proteins. RK, JD, RM, and AH analyzed bioinformatic data. KW, SP, and DM supervised the research. RK and KW wrote the manuscript. RK, JD, PB, DM, BF, RM, AH, SP, and KW revised the manuscript. All authors approved the final manuscript.

## Conflict of Interest

PB, JM, and DM were employed by the company Varigen Biosciences Corporation at the time of this work. No patents or products were developed from this work. The remaining authors declare that the research was conducted in the absence of any commercial or financial relationships that could be construed as a potential conflict of interest.

## Publisher’s Note

All claims expressed in this article are solely those of the authors and do not necessarily represent those of their affiliated organizations, or those of the publisher, the editors and the reviewers. Any product that may be evaluated in this article, or claim that may be made by its manufacturer, is not guaranteed or endorsed by the publisher.

## Footnotes

1
https://ftp.ncbi.nih.gov/pub/mmdb/cdd/little_endian/Cdd_LE.tar.gz

2
https://www.geneious.com
