# Supplementary material for: Novel Viral DNA Polymerases From Metagenomes Suggest Genomic Sources of Strand-Displacing Biochemical Phenotypes
Source: Front Microbiol. 2022 Apr 21;13:858366. doi: 10.3389/fmicb.2022.858366 (PMC9069017; doi:10.3389/fmicb.2022.858366)
Supplement: Supplementary file 5 [file Data_Sheet_1.docx]

Supplementary Material

## Supplementary Figures


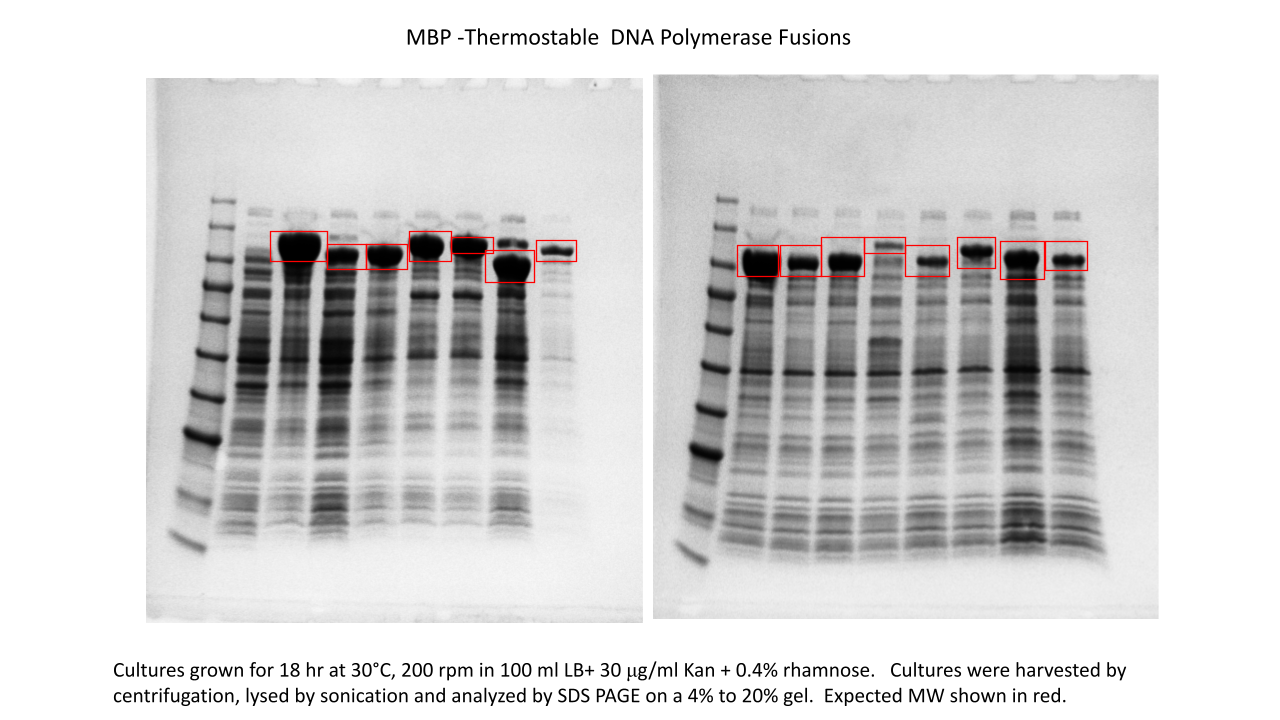


**Supplementary Figure S1. SDS-PAGE gels of biochemically inactive MBP-DNA polymerase fusions.** Cultures were grown for 18 h at 30 ℃, 200 rpm in 100 mL LB, 30 µg/mL kanamycin, 0.4% rhamnose.  Cultures were harvested by centrifugation, lysed by sonication, and analyzed by SDS‑PAGE on a 4–20% gradient gel.  Expected molecular weight outlined in red.
